# 

**Published:** 1916-06-17

**Authors:** 


### A Legal Quibble.

The war is now providing questions for our judges to settle with regard to the informal wills left by sailors and soldiers. The will of a "soldier being on actual military service or a mariner or seaman being at sea" does not require to be executed with the formality and solemnity of an ordinary will, but in order that the informality may be overlooked the conditions embodied in the statutory words quoted must be observed. How questions arise regarding these is well illustrated by a case lately decided by Mr. Justice Bargrave Deane, a case which deserves the attention of medical men or students who may be serving in any capacity in the war. In the case referred to the maker of the will in question, one Anderson, was a member of the Royal Naval Auxiliary Sick Berth Reserve of the St. John Ambulance Brigade, and being so he was not in the direct service of the Crown, having merely contracted with the St. John Brigade to serve for one year in the Navy if required. Anderson was called up for service by the Society on August 2, 1914, and received orders to report himself on board H.M.S. *Pembroke*, which is the naval name of Chatham Barracks. Anderson made his will the same day and on August 3 he reported in uniform at Chatham. He was drowned on October 30, 1914, in the loss of the hospital ship *Rohilla*. His will would, as the will of a person in civilian life, be invalid, but it was sought to set it up as the will of "a mariner or seaman being at sea." The Court held that Anderson was a seaman at the time the deed was made, but that he was not at sea; and therefore the document was not entitled to the privileges of a sailor's will. Had Anderson gone to sea, come back, and made his will when ashore on leave, it would, by the authority of an earlier decision, be valid, but at the date of the will he had never been to sea. The decision may seem arbitrary, but it is the duty of the Courts to see that only the statutory exceptions to formal and proper execution are permitted.

### HOSPITAL RESULTS IN 1915.

**Addenbrooke's Hospital, Cambridge.**— The annual report is most attractive in appearance, has a number of excellent illustrations, and is one that it is a pleasure to look through. But for reasons of economy the issue might have been printed on much thinner paper without losing any of its merits; especially does this remark apply to nearly 100 pages of subscribers' names. Although 487 soldiers have been admitted, the total number of civil patients treated in the wards was about the same as in the previous year; the number of out-patients has fallen from 9,903 to 9,157. The total income (exclusive of legacies, £4,500) was £11,408, and the total expenditure £12,300. From the districts served by the hospital 13,000 eggs have been contributed, but the former considerable support in gifts of fruit and vegetables has fallen off.

**Birmingham and Midland Eye Hospital.**—There was an increase of 299 in-patients. One-third of the 108 beds were set aside for soldiers, and 362 were transferred from the military hospital. There was an increase of 2,755 in the number of out-patients treated, due almost entirely to casualty cases, 95 per cent. of whom were engaged on munition work. Total income (including £421 legacies), £9,019. Total expenditure, £8,825.

**Bolton Infirmary and Dispensary.**—Mr. Alderman Nicholson, who died last year, had been a member of the committee for fifty-three years, chairman since 1896, and treasurer since 1885. He is succeeded as chairman by Mr. Percy Musgrave, and as treasurer by Mr. J. H. Bradbury. One hundred and thirty-two military cases had been admitted up to the end of February. Bolton Infirmary is one of the few important institutions in this country that do not make up their accounts from January to December. It would be a very simple matter to come into line at the present time, when one year's figures are less comparable with those of another than in the even times of peace.

**Ear and Throat Hospital, Birmingham.**—There was a decrease in the number of in-patients treated, but the average length of stay was 50 per cent. more. The number of out-patients was greater than in 1914. Total income (including £718 legacies), £4,080. Total expenditure, £3,865.

**St. John's Hospital, Lewisham.**—The beds have been increased from forty-six to sixty to provide for soldiers, 199 of whom were received last year. Out-patient numbers decreased, but the attendances were considerably more, partly accounted for by inoculations of soldiers before proceeding on service. Total income (including legacies £530), £4,426. Total expenditure, £4,037. Estimated value of gifts in kind, £113.

**Worcester General Infirmary.**— There was an increase of 207 amongst in-patients and a decrease of 210 amongst out-patients. Wounded soldiers (number not stated) have been received. The average cost per occupied bed has fallen from £81 to £72, but the average cost of each in-patient has risen. The cost of provisions, divided by the daily average number of resident staff and patients, amounted to £16 17s. 1d. per head, as against £15 16s. 1d. Subscriptions have fallen by £85.

### PASSING EVENTS AND TOPICS.

### A Dental Clinic for a Military Hospital.

At a cost of £1,000 a dental clinic is being prepared in connection with the 2nd Northern General Hospital, Leeds.

### Residential Sanatorium School.

A residential sanatorium school at Castleshaw has been opened by the Oldham Education Committee, Mrs. C. E. Lees, of Werneth Park, having defrayed the cost of the necessary additions and equipment.

### Maternity and Child Welfare.

The Hyde Town Council has adopted a maternity and child welfare scheme, and decided to appoint a lady doctor to act under the Education and Child Welfare Committees at a salary of £350 per annum.

### Vaccinating an Entire Population.

According to reports in the German medical Press, the entire population in Galicia is being vaccinated. The work is being done by students from the Universities of Cracow and Lemberg, who have been released from their studies for six weeks. The students receive free lodging and eight shillings a day each.

### A Factor in Hospital War-Time Expenditure.

One of the effects of the war on some hospitals has been the necessity for paying fees to resident staffs who formerly gave their services free. For instance, Westminster Hospital, which, being a hospital with a school attached, had before the war a regular staff of residents who received no payment, is now paying its staff twenty guineas a week. This is an extra expense which it is hoped the generous public will bear in mind.

### Duchess of Marlborough as Wall-Breaker.

The West Ham Hospital having, through the generosity of Sir William and Mr. Edwin Tate, acquired the adjoining St. John's Vicarage, the Duchess of Marlborough inaugurated a home for the nurses of the hospital by knocking down a portion of the dividing wall with a mason's mallet.

### Local Hospital Statistics.

In Manchester and Salford institutions it is claimed that one accident case is dealt with every 8½ minutes of the day and night, making a total of 61,931 such cases for the year. During 1915 there were 29,978 in-patients, 457,230 out-patients, and 13,963 home-patients. The operations performed numbered 65,693.

### Gifts of Ambulances.

Eleven motor-ambulances, costing £4,345, have been presented to the Scottish branch of the British Red Cross Society by the drapery trade in Glasgow and district, whilst the Liverpool branch of the Red Cross have received a motor-ambulance, purchased with money raised by Liverpool school-children collecting and selling medicine bottles.

### The London Spectacle Mission Society.

The useful function of this Society in supplying "spectacle cards" on the same principle as hospital "letters" was carried on successfully last year, although the operations of the Society were somewhat affected by the war. The annual report states that 3,000 needy persons suffering from impaired vision were helped to provide themselves with suitable glasses.

### MATERNITY AND CHILD WELFARE.

The Royal Sanitary Institute offer a prize of £50 and the medal of the Institute for the best thesis, setting out a complete and practical scheme for maternity and child welfare work, suitable for adoption by local authorities. The following, amongst other points, should be dealt with:—Ante-natal care and the means of exercising it; child welfare from birth to school age; formation and functions of clinics or other suitable institutions; control in the home; home and institutional instruction and treatment; the utilisation of existing organisations or agencies; the qualifications and payment of officers and workers. Application for general conditions should be made to the Secretary of the Institute, 90 Buckingham Palace Road, London, S.W

### Matrons' and Sisters' Appointments.

HINCKLEY INFECTIOUS DISEASES HOSPITAL.—Miss Teresa Dowling has been appointed matron. She received her general training at the Township of South Manchester Hospitals, and her fever training at the City Fever Hospital, Parkhill, Liverpool. She has since been matron at Hawarden Isolation Hospital.

NORTH END INFECTIOUS DISEASES HOSPITAL, EASTLEIGH.—Miss Mary Lloyd has been appointed nurse-matron. She was trained at the Prince of Wales's General Hospital, Tottenham, has held appointments at Bradford and at Sunderland Royal Infirmaries, and has been deputy-superintendent and senior charge nurse at Farnham Infirmary.

BIERLEY HALL SANATORIUM FOR TUBERCULOSIS, BRADFORD.—Miss Ethel Hartley has been appointed sister. She was trained at Townley's Hospital, Bolton, and has since held various appointments and done private nursing.

DANYCOED RED CROSS HOSPITAL, BLACKPILL, GLAM.—Miss M. C Cameron has been appointed sister. She was trained at Bristol Royal Infirmary, has held the post of sister at Government hospitals in the Transvaal and the Straits Settlements, been staff nurse at the 2nd Southern General Hospital, Bristol, and sister-in-charge at Cottesmore Red Cross Hospital, Haverfordwest.

DR. BARNARDO'S HOME, BIRKDALE.—Miss Marjorie Cox has been appointed sister. She was trained at the East Sussex Hospital, Hastings.

HATHERSAGE RED CROSS HOSPITAL, DERBYSHIRE.—Miss Gertrude M. Cook has been appointed night sister. She was trained at Sheffield Union Hospital, where she has since been sister, and has also done private nursing. Miss Cook holds the certificate of the Central Midwives Board.

HOSPITAL OF ST. CROSS, RUGBY.—The Hon. Felicie Norton has been appointed night sister. She was trained at Kidderminster Infirmary, and has since been night sister at the Royal Victoria and West Hants Hospital, Bournemouth, and a sister of Queen Alexandra's Imperial Military Nursing Service.

ROYAL GWENT HOSPITAL, NEWPORT (MON.).—Miss A. M. Evans has been appointed sister. She was trained at the Royal Gwent Hospital, where she has since been theatre and ward sister.

PRESTON ROYAL INFIRMARY.—Miss Mary S. Allan has been appointed ward sister. She was trained at Huddersfield Royal Infirmary, and has since been sister at Stockton and Thornaby Hospital, and night sister at Coventry and Warwickshire Hospital.

PUTNEY GENERAL HOSPITAL.—Miss E. M. Palmer has been appointed sister. She was trained at Chelmsford and Essex Hospital, and has since been staff and charge nurse at Nuneaton General Hospital and theatre sister at Watford General Hospital.

ROYAL WATERLOO HOSPITAL.—Miss Elizabeth Annie O'Brien has been appointed night sister. She was trained at St. Bartholomew's Hospital, Rochester, and at the Eastern Fever Hospital, Homerton, and has been ward sister at the South-Western Fever Hospital, Stockwell.

**NOTE.—Particulars of Appointments for insertion in this column will be welcomed.**

### A Bursary for Medical Women.

IN memory of the late Dr. Mary Murdoch, of Hull, it is desired to establish a bursary to help some young medical women in the first years of their practice in the School of Medicine for Women at the Royal Free Hospital. The committee formed for this purpose includes several women doctors, in addition to Miss L. B. Aldrich Blake, M.D., M.S., dean of the school.

### The Marine Society.

MANY members of the staff of the training-ship *War-spite* have been called up for active naval service, and of the list of officers and petty officers published in the Marine Society's Report for 1915 more than half are marked as temporary officers during the war. During the 159 years of the Society's work 67,178 trained lads have been sent to sea, and of this number 29,143 have joined the Navy. Last year's recruits to the Merchant Service are earning from £3 to £5 a month as ordinary seamen and £2 to £3 as deck-boys; these wages are much in excess of pre-war periods. The expenditure of the Society during 1915 was £12,333, about 64 per cent. of which was met by the revenue from invested funds.

Rates of Subscription: Twelve months, 6/6; Six months, 4/-; Three months, 2/-, post free to any address in the United Kingdom. Abroad, Twelve months, 8/8; Six months, 5/-; Three months, 2/6, post free.—Address The Subscription Dept., "The Hospital," The Hospital Building, 28/29 Southampton Street, Strand, London, W.C.

